# A case of long-term relief from bleeding due to fungating breast cancer in an elderly patient after 8-Gy single-fraction radiation therapy

**DOI:** 10.1007/s13691-025-00783-w

**Published:** 2025-06-30

**Authors:** Yumi Kokubo, Ryo Ashida, Peter J. K. Tokuda, Takamasa Mitsuyoshi, Toshiyuki Imagumbai, Masaki Kokubo

**Affiliations:** Department of Radiation Oncology, Kobe City Medical Center General Hospital, 2-1-1 Minatojima Minamimachi, Chuo-Ku, Kobe, Hyogo 650-0047 Japan

**Keywords:** Breast cancer, Palliative care, Care of elderly, Single-fraction radiation therapy

## Abstract

Fungating lesions in advanced breast cancer often affect the quality of life. Fractionated radiation therapy is the preferred treatment over single-fraction radiation therapy because of the relatively favorable prognosis for breast cancer. Therefore, limited literature is available regarding the effectiveness of short-course radiation therapy, especially 8-Gy single-fraction radiation therapy. Herein, we describe the case of an 85-year-old patient diagnosed with locally advanced breast cancer for several years who underwent two sessions of 8-Gy single-fraction radiation therapy performed approximately one year apart. This therapy resulted in prolonged control of bleeding without severe side effects. The patient experienced bleeding from a fungating lesion and was referred to the Department of Radiation Oncology for palliative therapy to relieve the bleeding. Owing to limited support from her family and nursing care workers, she faced transportation challenges making frequent hospital visits infeasible, so 8-Gy single-fraction radiation therapy was performed. Radiation therapy was well tolerated, and hemostasis was achieved. Eleven months later, tumor regrowth and recurrent bleeding occurred, which necessitated another 8-Gy therapy. Re-irradiation was tolerated with only mild dermatitis noted, and the symptoms were relieved. Twelve months after re-irradiation, the breast cancer remained controlled, with no further bleeding. These findings indicate that 8-Gy single-fraction radiation therapy effectively controls bleeding from fungating breast cancer, and hemostasis may last longer than previously reported. Moreover, this method may be a valuable way to provide symptomatic relief, especially for elderly patients needing nursing care, even when their prognosis is relatively good.

## Introduction

In advanced breast cancer, fungating lesions significantly affect quality of life [1]. Bleeding from fungating lesions is commonly observed and palliative radiation therapy is sometimes used to address this issue [2]. Fractionated radiation therapy is the preferred treatment for breast cancer because of its relatively favorable prognosis [3–10]. However, it may not be suitable for patients with a poor performance status, such as the elderly. There is limited literature available on the effectiveness of short-course radiation therapy, especially 8-Gy single-fraction radiation therapy (SFRT). Here, we report the case of an elderly patient with breast cancer, residing in an institution and facing challenges in visiting the hospital, who experienced prolonged control of bleeding without significant side effects. This was achieved through two sessions of 8-Gy SFRT performed approximately one year apart.

## Case report

**Fig. 1:**
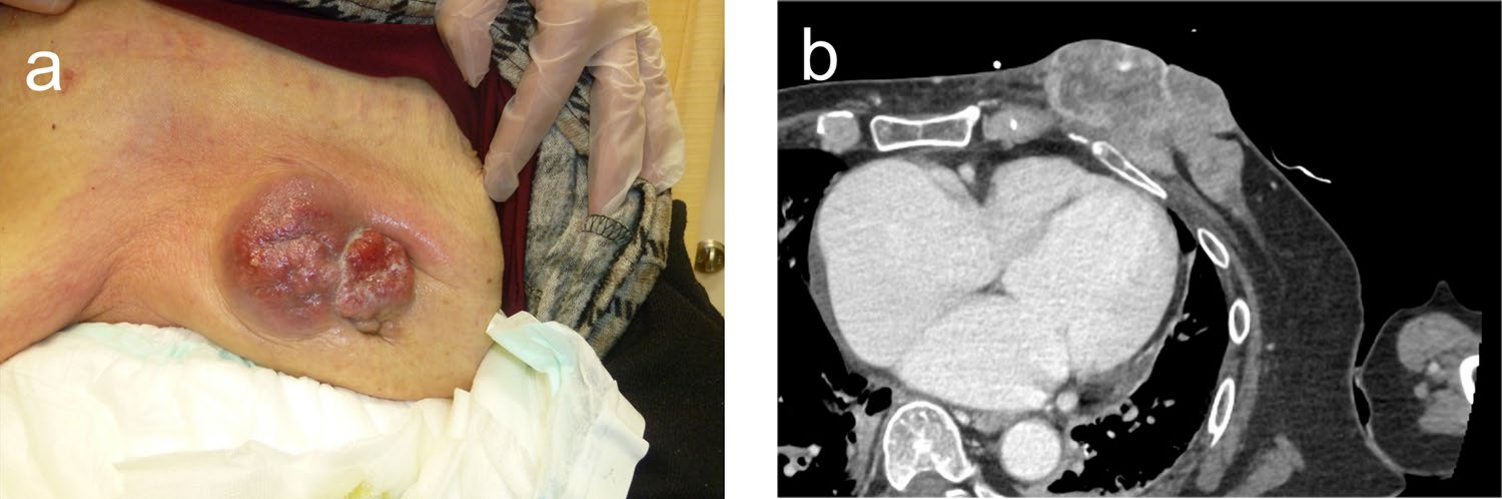
Initial presentation of the breast mass. **a** Physical examination revealed a 60 × 50 mm nodular mass occupying the left breast, accompanied by surrounding erythema. **b** A computed tomography (CT) scan showed a centrally necrotic, peripherally contrast-enhanced soft tissue mass centered in the left breast

**Fig. 2:**
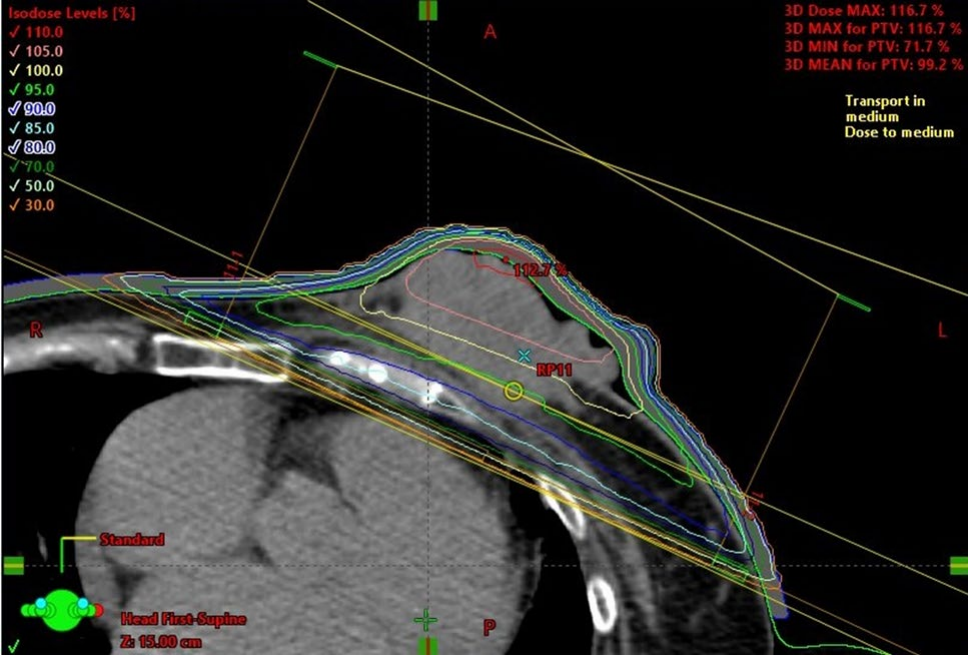
Representative axial CT slice from the first treatment, showing the distribution of the radiation isodose line

**Fig. 3:**
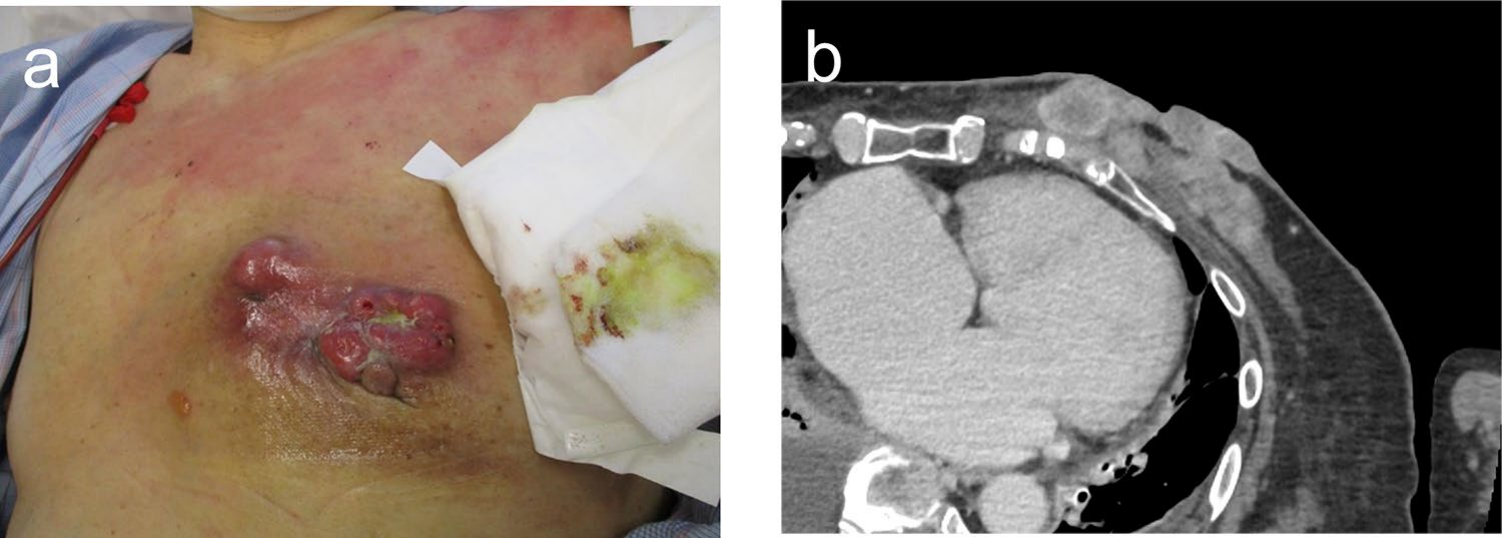
Response of the breast mass to radiation therapy. **a** Regression of the mass at four months post-treatment, observed on physical examination. **b** CT scan at 4 months following the completion of radiation therapy

**Fig. 4:**
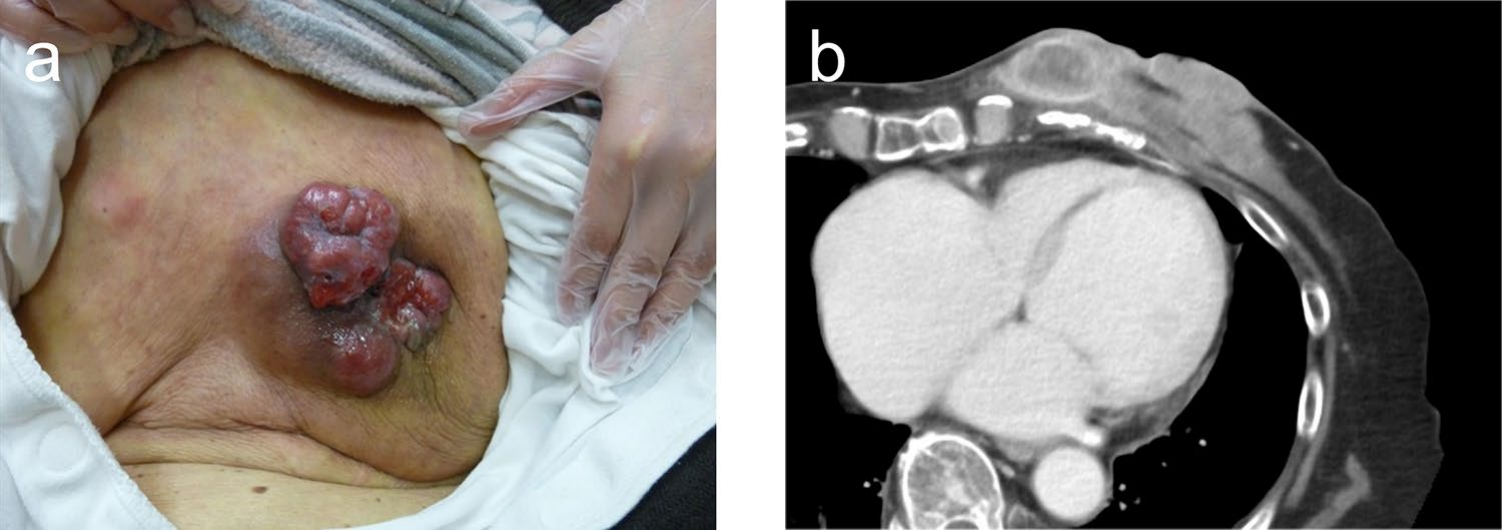
**a** A physical examination and **b** a CT scan have revealed the recurrence of the breast mass, which was observed 11 months following the completion of radiation therapy

**Fig. 5:**
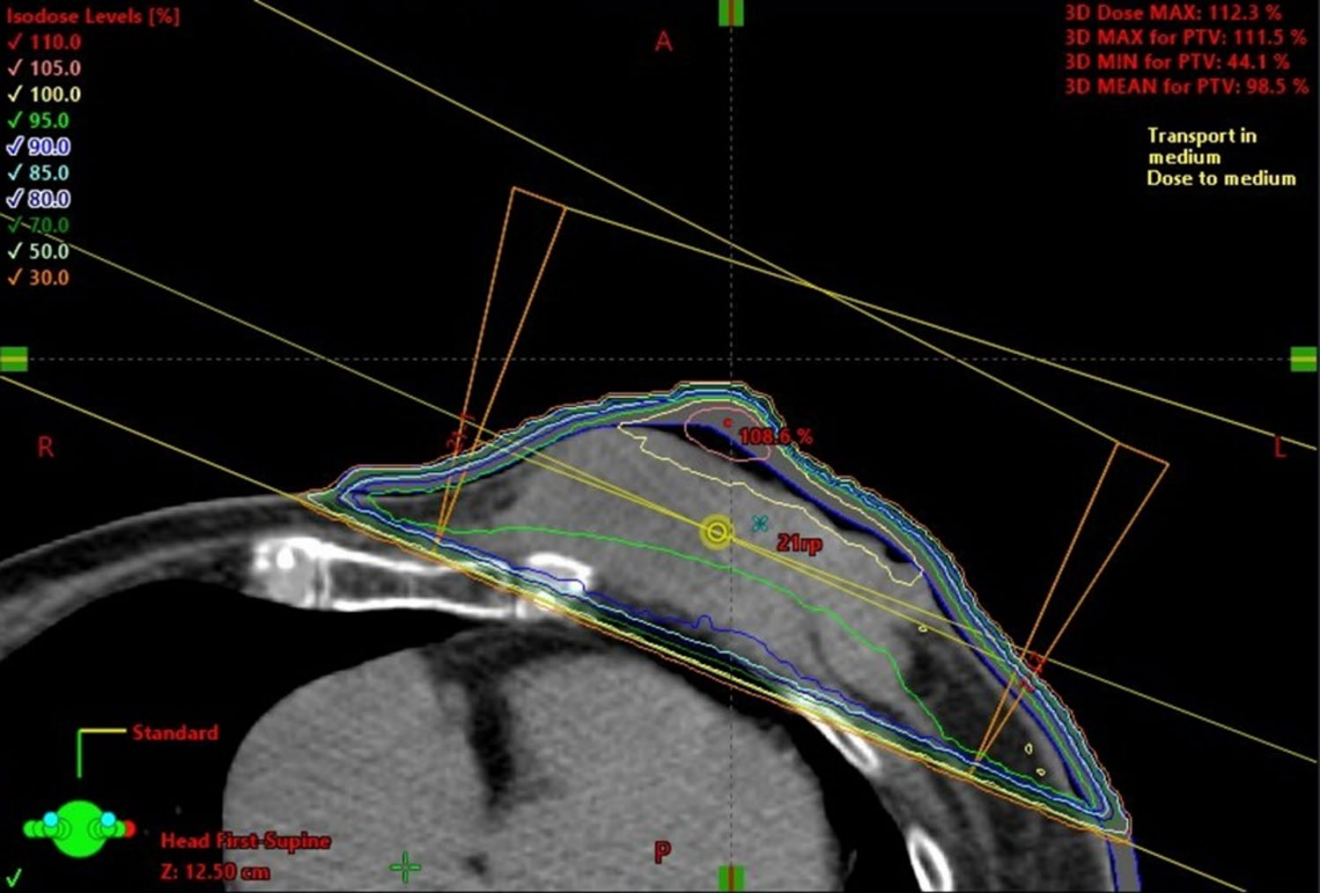
CT-based plan at the time of re-irradiation, illustrating the use of wedges to ensure dose homogenization

**Fig. 6:**
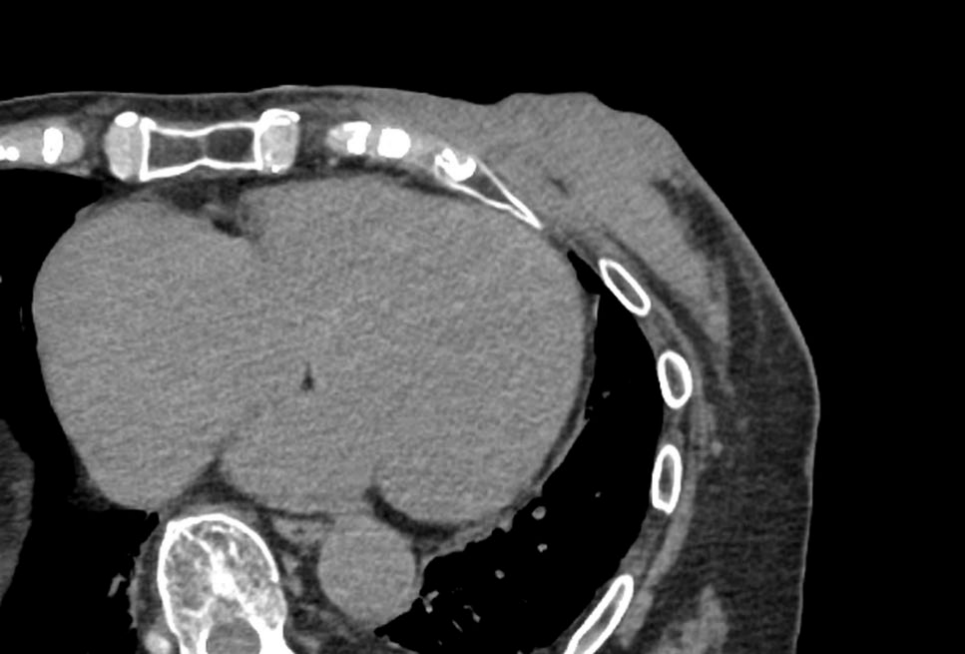
CT scan at eight months after the completion of re-irradiation

An 85-year-old woman presented to the Department of Breast Surgery in December 20XX with a three-month history of a fungating lesion on her left breast. Due to her history of cerebral infarction and dementia, she had difficulty communicating. Her Eastern Cooperative Oncology Group (ECOG) performance status was 4. She required intensive nursing care and had been living in a care home for several years. The breast mass had been noticed by her family and nursing care workers a couple of years prior, and they had been monitoring it since no symptoms were observed. Physical examination revealed a nodular mass in the left breast, with surrounding erythema (Fig. 1a). A core needle biopsy confirmed an invasive ductal carcinoma (Nottingham Grade 2, with estrogen receptor 100% positive, progesterone receptor negativity, and human epidermal growth factor receptor 2 negativity). Computed tomography (CT) revealed a soft tissue mass centered in the left breast (Fig. 1b) and metastasis to the axillary lymph nodes, but no distant metastases. Her family opted not to pursue active treatment, and a wait-and-see approach was adopted. However, one month later, she experienced bleeding from the fungating lesion. Her family was informed about hormonal therapy, but they preferred to explore alternative options for alleviating her symptoms. They were concerned about the need for regular hospital visits to monitor adverse events and obtain prescriptions. Therefore, she was referred to the Department of Radiation Oncology for palliative radiation therapy to relieve the bleeding. Owing to limited support from her family and nursing care workers, 8-Gy SFRT was performed. A CT-based treatment plan was used. The irradiation plan consisted of tangential pair fields using a 6-megavolt X-ray beam with a 5-mm bolus. Only the clinically or radiographically involved areas were irradiated, and the locoregional lymph nodes were omitted (Fig. 2). Radiation therapy was well tolerated with no observed side effects. One month after the treatment, the bleeding stopped. Tumor regression was observed four months later (Fig. 3a, b). However, 11 months later, tumor regrowth and recurrent bleeding occurred (Fig. 4a, b). The family received another explanation about hormonal therapy, but they remained firm on their wish to minimize hospital visits. As a result, they requested re-irradiation. After considering their wishes, 8-Gy SFRT was applied again. The irradiation techniques were similar to the first treatment, except wedge filters were used to ensure dose homogenization, given that it was a re-irradiation procedure (Fig. 5). The treatment was tolerated with mild normal tissue complications, and the tumor growth was suppressed 8 months after re-irradiation (Fig. 6). Twelve months after re-irradiation, the breast cancer remained controlled, with no further bleeding. According to the medical information letter, there was no re-bleeding at 12 months after the re-irradiation, and the patient was confirmed to be alive.

## Discussion

This case report describes two important clinical findings. Eight-Gy SFRT effectively controls bleeding from fungating breast cancer, and hemostasis may last longer than previously reported [9]. This method may be valuable in providing symptomatic relief, especially for elderly patients requiring nursing care, even when their prognoses are relatively good.

Eight-Gy SFRT has been found to effectively control bleeding from fungating breast tumors and may maintain hemostasis for a longer duration than previously thought [9]. Some reports have indicated that short-course palliative radiation therapy for locally advanced breast cancer is effective, especially for hemostasis. The only two available prospective studies demonstrated the effectiveness of fractionated radiation therapy, such as 36 Gy in 12 fractions [3, 4]. In most other available retrospective analyses, fractionated doses of 26–55 Gy have been reported to be effective [5–7]. Vempati et al. reported no clinical benefit of palliative radiation therapy at total doses of less than 30 Gy [8]. Conversely, Jacobson et al. noted no difference in efficacy between 8-Gy SFRT and fractionated therapy [9]. However, Jacobson et al. also found that 8-Gy SFRT did not provide long-term benefits and re-irradiation was often required, with a median time to re-irradiation as short as 2 months [9]. In the current case, re-irradiation was necessary, as in previous reports; however, hemostasis persisted for 11 months after the first irradiation and for 12 months after re-irradiation. Existing literature on the hemostatic effect of 8-Gy SFRT is limited, and this case suggests further potential for its effectiveness.

Eight-Gy SFRT may be a valuable palliative treatment for bleeding from aggressive breast cancer, particularly in elderly patients requiring long-term care. The median age at breast cancer onset has been increasing associated with an aging population in developed countries [11]. Those in institutionalized settings face challenges in accessing palliative care [12]. The treatment options for this condition include dressings, medication (commonly topical agents), surgery, interventional radiology, and radiation therapy [13]. Surgery and interventional radiology are sometimes too invasive. While the application of dressings and topical agents, such as Mohs’ paste, is less invasive, it typically requires ongoing care and may warrant frequent hospital visits unless radical treatment is provided. In contrast, 8-Gy SFRT stands out as a minimally invasive option that reduces the need for hospital visits and eases caregiver burden. Additionally, it offers fundamental treatment for tumors. Overall, 8-Gy SFRT is well-balanced in every aspect and might be considered a suitable option for elderly patients.

The hemostatic effects of SFRT have been reported in various types of cancers, including gastric, pancreatic, bladder, and cervical cancer [14–17]. As symptom relief with SFRT is not thought to be long lasting, it is commonly recommended for patients with a particularly poor prognosis [15–17]. Sapienza et al. reported that short regimens, including 8-Gy SFRT, and longer regimens demonstrated equal durability; however, few patients with breast cancer were included in the study [18]. Breast cancer, which generally has a better prognosis, is typically treated with fractionated radiation therapy [9]. In this case, the tumor was localized, and the prognosis was favorable; therefore, fractionated irradiation was considered first. However, given the difficulty of making frequent hospital visits, 8-Gy SFRT was performed. Although re-irradiation was necessary, it was performed safely and resulted in long-term symptom control with a minimal burden. Since 8-Gy SFRT may control symptoms for a more extended period than previously reported, 8-Gy palliative radiation therapy should be considered in elderly patients who require nursing care, regardless of their prognosis.

In conclusion, 8-Gy SFRT effectively stopped bleeding from fungating breast cancer for a longer period than would have been previously expected. This treatment can be especially helpful in providing relief to elderly patients who need nursing care, regardless of their prognosis. Historically, this treatment option has not been widely considered for palliative care of patients with breast cancer. However, when addressing bleeding in elderly patients with advanced breast cancer, 8-Gy SFRT should be considered as a promising option. As more cases are studied, the effectiveness of 8-Gy SFRT in combination with drug therapy should be explored.

## Data Availability

Not applicable.
